# Tracing the bounds of distress: Mental health and the Lancet Commission on lessons for the future from the COVID-19 pandemic

**DOI:** 10.7189/jogh.14.03022

**Published:** 2024-05-17

**Authors:** Anthony Maher

**Affiliations:** Department of Psychiatry, University of Toronto, Ontario, Canada

With the aim of exploring the mental health implications of the Lancet Commission on lessons for the future from the coronavirus disease 2019 (COVID-19) pandemic, in this paper, seven key themes related to mental health that emerge from the Commission report are presented. I contend that these themes are explicitly or implicitly acknowledged within the Commission’s findings.

Through integrating observations from recent policy and research developments in this space as well as clinical practice guidelines, I offer a perspective on the potential strengths and limitations of the Commission’s observations on mental health.

## A CONCEPTUAL FRAMEWORK FOR MENTAL HEALTH

First, within the initial conceptual framework entitled ‘Five pillars of the successful fight against emerging infectious diseases,’ mental health is explicitly mentioned in the third pillar as follows: ‘The third is health services: to save the lives of people with the disease and ensure the continuity of other health services, including those for mental health’ [1]. This establishes at the outset that mental health is integral to and inseparable from health services in the context of the pandemic. Using general language in this framework (i.e. ‘those for mental health’), one can imagine that the authors are trying to capture the immense variability in mental health resources on a global scale. To this end, it is worth underscoring the stark differences between global regions in terms of access to psychiatric beds in care settings, with data showing the European region having a median of eight beds per 10 000 population [2], compared to Africa having only 0.34 beds per 10 000 population [3].

Mental health care is also specifically included in section three of the report, which is related to recommendations regarding ensuring care for pandemic-related and non-pandemic-related issues. These recommendations further include advocacy for social support in areas such as gender-based violence and indigency. This implies an understanding of mental health that includes the social determinants of health [4]. Of note, this approach is also broadly consistent with the 2021 version of the World Health Organization’s (WHO) Comprehensive Mental Health Action Plan 2013–30 [5], such as objective three and related sub-targets on implementing strategies for promotion and prevention in mental health.

## MENTAL HEALTH WORKFORCE

Beyond the conceptual framework, the report refers to ‘investment in a skilled workforce’ as an essential component of national pandemic preparedness plans. Although mental health is not specifically mentioned in this context, the authors note, for instance, that people who could work online during the pandemic fared better regarding health and socioeconomic well-being than those in goods-producing industries or essential workers, such as health care workers. They observe that these latter individuals are often required to attend work in person. It follows that health services – including mental health services – cannot exist without a skilled workforce. Recent studies have proposed strategies such as shift duty schedules and burnout prevention programmes as potential ways to support health workers [6]. The mental health impacts of the pandemic on health care workers arguably necessitate the development of such strategies. This is further supported by emerging evidence, as nearly half of respondents in a United States survey of health care workers reported serious psychiatric symptoms, including suicidal ideation, in the context of the COVID-19 pandemic [7].

## PROSOCIAL BEHAVIOUR

Third, the authors support the promotion of ‘prosocial’ behaviour for mental and physical health. This behaviour is defined as ‘the orientation of individuals and government regulations to the needs of society as a whole, rather than to narrow individual interest.’ This may include a range of individual and/or collective behaviours, such as wearing face masks at the individual level or enforcing workplace safety standards at the level of government decision-makers. The Commission argues for this approach ‘both during pandemics and beyond.’ This implies a role for prosocial behaviour during pandemic times and as a form of prevention of mental illness – even during non-pandemic times.

We can start with behaviour during pandemic times. The authors note that governments must support people in making prosocial behavioural changes, such as self-isolating when necessary. However, from a psychiatric perspective, it is challenging to imagine how individuals facing daily news reports about the growing spread of a potentially deadly virus ought to manage the very real threats to their own individual well-being while simultaneously being mindful of the needs of society as a whole. Indeed, this is a big ask. When considering the impacts of asking an individual living with a severe mental illness to act more ‘prosocially’ it becomes even more challenging to map out how this ‘prosocial’ orientation can be fostered in a practical, actionable manner.

Furthermore, how this might look outside the context of a pandemic is arguably even less clear. Using the individual level of analysis proposed by the authors, how might trust in institutions be fostered across contexts and societies and in a lasting manner? How is trust fostered between a citizen and their government at any point? This is a complex question and brings in issues related to cultural and socio-political differences, particularly when one considers the impacts of living in countries that have faced longstanding corruption and mistrust between citizenry and government [8]. This point is not fully addressed in the Commission’s report. In other words, finding ways to decrease the likelihood of adverse mental health outcomes during non-pandemic times seems to fall outside the scope of the report.

## INDIVIDUAL VS POPULATION-LEVEL CONSIDERATIONS

Fourth, the Commission draws attention to potential differences between individual and population-level thinking related to mental health. They note that population-level trends may mask the distress felt by certain individuals and that individuals with pre-existing mental health conditions have continued to live with these during the pandemic.

Returning to the example of prosocial behaviour, such as wearing a face mask to protect oneself and others from viral spread, one could consider the impact of comorbid mental illness on the ability of individuals to adopt such behaviours consistently over time. For example, in cases where an individual living with bipolar disorder is experiencing a manic or hypomanic episode and exhibits core symptoms of this phase of illness (such as distractibility), he or she may not be consistently able to comply with regulations such as the wearing of a face mask. Therefore, how one might take such a recommendation and put it into practice is an open question that the report does not directly address. To the extent that clinicians such as psychiatrists and family physicians typically see individual patients, this acknowledgement also speaks to the reality of clinical decision-making in mental health.

## DIGITAL SERVICES

Fifth, the authors acknowledge that universal access to digital services is vital, particularly in emergency services. However, the expansion of digital or virtual delivery of mental health care is not specifically discussed in the Commission report, and this is despite others, such as the Lancet World Psychiatric Association Commission on Depression, observing that digital technology has become a ‘standard delivery platform for mental health care’ [9].

Given the extent of this shift, it seems prudent to reflect further on the implications of expanded virtual care delivery. There is a growing recognition of the need to study the impacts and effectiveness of digital interventions during the COVID-19 pandemic [6]. Although there are potential implications for population-level practices such as surveillance, implications at the individual level must also be considered. For example, existing clinical practice guidelines may lag behind real-world practice, and virtual care is no exception. In one such guideline from Canada, internet-based cognitive-behavioural therapy is described as ‘a newer treatment that may increase the availability of cognitive-behavioural therapy for anxiety and mood disorders in the future’ [10]. This is despite the fact that the use of virtual platforms in the delivery of psychiatric care has exploded since the onset of the pandemic and, in many settings, now forms a routine part of mental health care delivery. We have seen, in the words of one group of psychiatrists from Canada, ‘the meteoric rise of telepsychiatry’ [11]. Several concerns have been identified related to this rise. Potential barriers to the uptake of virtual psychiatry services have been highlighted, such as the ability of users of digital technologies, competency of providers, as well as cultural appropriateness concerns [12]. While these concerns are of ever-growing importance, the Commission’s authors do not explicitly address them.

## REFLECTING ON ‘LONG COVID’

Sixth, the Commission reports that long COVID has ‘substantial physical, mental, social, and economic effects, and long COVID might itself be an emerging pandemic.’ This raises the question: how might we implement care for these potentially substantial needs in the context of the immense existing pressures on health systems, both with respect to mental and physical health care? The Commission acknowledges this complexity, noting that multidisciplinary and stigma-free care is unavailable in many settings.

At the same time, it must be emphasised that there is ongoing uncertainty regarding the long-term prognosis of people who have recovered from COVID-19 [6], particularly with respect to neuropsychiatric outcomes [13]. The Commission report is variable in its references to long COVID, and this represents a limitation. For instance, they opine that ‘high infection rates mean high rates of long COVID’ and link this to ‘physical and mental suffering to individuals and their families,’ while later taking a more conditional approach, stating that ‘as COVID-19 is likely to become endemic, many people could have long-term health care and social care needs.’ This latter approach seems to be echoed in recent research, with one systematic review from 2022 showing that while the overall effect of the pandemic has been linked with worsening psychiatric symptoms, the long-term effect from direct COVID-19 infection has been associated with no or mild symptoms [14]. Indeed, the ever-changing nature of this research cannot be overemphasised. This has direct relevance for clinicians in mental health care as well. To the extent that individual patients routinely ask care providers about the links between COVID-19 and mental health, front-line clinical guidance around these potential links remains in a state of flux.

## DEFINING PSYCHOPATHOLOGY WITHIN AND BEYOND PANDEMICS

Finally, the understandings of mental illness that are implicitly espoused in the Commission’s report are worthy of exploration. As an example, the report refers to ‘long-term disabilities arising from long COVID and from the mental distress of the pandemic.’ This raises a possible tension between ‘mental distress’ on the one hand and psychiatric pathology on the other. A further example is in the section on school closures during the pandemic, with the authors noting ‘devastating effects on student learning, mental health, socioemotional outcomes and lifetime earning potential.’ How might we differentiate mental health from socioemotional outcomes? Does one suggest a greater need for intervention than the other?

It is unclear in these instances and throughout the report whether the references to mental health capture responses that are within the realm of normal human experience or whether they amount to mental disorders, as captured by resources such as the Diagnostic and Statistical Manual of Mental Disorders [15]. It is inaccurate to conclude that each and every individual who experienced feelings of anxiety, trepidation, or uncertainty during the pandemic met the diagnostic criteria for a clinically significant mood or anxiety disorder. Indeed, emotional responses such as frustration or loss can be appropriate depending on the situation at hand, and often, it is the role of mental health professionals to help delineate such responses from clinically significant psychopathology. It follows that research has a critical role in delineating normal from abnormal responses to pandemics and tailoring responses to both [6]. A pledge to double the global output of mental health research is therefore reflected in the updated targets put forth by the WHO in its mental health action plan [5].

The Commission does not propose specific mechanisms for promoting this kind of research. As a possible model for research and/or translation between policy and clinical practice for mental health presentations that may be related to COVID-19, one might consider the conditions for further study in the Diagnostic and Statistical Manual of Mental Disorders. As the American Psychiatric Association states in its description of conditions included in this section: ‘Some proposed conditions had clear merit but ultimately were judged to need further research before they might be considered as formal disorders.’ This is based on ‘the amount of empirical evidence available on a diagnosis, diagnostic reliability or validity, a clear clinical need, and potential benefit in advancing research’ [16].

## CONCLUSIONS

In conclusion, in this paper, I aimed to highlight key thematic areas that relate to mental health in the Commission’s report and raise dilemmas that may be the focus of current and future inquiry. Several avenues for further research emerge from this report and the discussion introduced above. These may include research on potential mechanisms to foster trust in public institutions related to health, the mental health impacts of long COVID, as well as ways to bridge the gap between clinical practice and research related to mental health during pandemics. While the main purpose of this paper is not to detail new guidelines or policy recommendations, it is clear that the Commission report may be a useful platform for creating responses to potential future pandemics and improving our current responses to the remnants of the COVID-19 pandemic. The thematic areas identified in this paper may be one way to organise such responses, such as those related to virtual mental health care, individual vs population-level considerations, and human resource planning for mental health professionals.
